# Reducing Friction in Orthodontic Brackets: A Matter of Material or Type of Ligation Selection? In-Vitro Comparative Study

**DOI:** 10.3390/ma15072640

**Published:** 2022-04-03

**Authors:** Anca-Oana Dragomirescu, Maria-Angelica Bencze, Adriana Vasilache, Elina Teodorescu, Cristina-Crenguța Albu, Nicoleta Olivia Popoviciu, Ecaterina Ionescu

**Affiliations:** 1 Department of Orthodontics and Dentofacial Orthopaedics, Faculty of Dental Medicine, “Carol Davila” University of Medicine and Pharmacy, 020021 Bucharest, Romania; anca.dragomirescu@umfcd.ro (A.-O.D.); adriana.vasilache@umfcd.ro (A.V.); elina.teodorescu@umfcd.ro (E.T.); olivia.popoviciu@umfcd.ro (N.O.P.); ecaterina.ionescu@umfcd.ro (E.I.); 2 Department of Genetics, Faculty of Dental Medicine, “Carol Davila” University of Medicine and Pharmacy, 020021 Bucharest, Romania

**Keywords:** dentistry, orthodontics, frictional force, bracket, ceramics, self-ligation

## Abstract

(1) Background: Orthodontic appliances have changed and improved with the increasing demand for orthodontic treatment of the general population. Patients desire for shorter orthodontic treatments and for the wearing of more aesthetic devices has led to the technological development of orthodontic brackets; these were manufactured from aesthetic materials (ceramics, composite polymers) and presented different designs regarding the way archwires are ligated to the bracket. The aim of this study was to determine whether there were any differences between the static frictional forces generated by stainless steel (metallic) and polycrystalline alumina (ceramics) conventional and self-ligating brackets. (2) Methods: Static friction assessment was carried out in vitro with a universal testing machine, HV-500N-S (Schmidt Control Instruments, Hans Schmidt & Co. GmbH), intended for measuring compression and traction forces. (3) Results: The study revealed significant differences in static frictional forces at the bracket-archwire interface between the tested brackets. Stainless steel brackets produced lower static friction forces than polycrystalline alumina and self-ligating brackets generally produced lower static frictional forces than conventional brackets. The reduction of frictional forces was noticeable in the first stages of treatment, when thin, flexible orthodontic archwires (0.016” NiTi) are used. Engaged with large rectangular stainless steel archwires, (0.019 × 0.025” SS), the frictional forces produced by conventional and self-ligating metal brackets were similar, no significant differences being observed between the two types of metallic design. However, in the case of tested ceramic brackets, the results showed that the self-ligating type allows a reduction in frictional forces even in advanced stages of treatment compared to conventionally ligation. (4) Conclusions: From the perspective of an orthodontic system with low frictional forces, metal brackets are preferable to aesthetic ones, and self-ligating ceramic brackets are preferable to conventional ceramic brackets.

## 1. Introduction

Orthodontic therapy with fixed appliances using the straight-wire technique has gained popularity nowadays due to its advantages, including a shorter treatment time, greater comfort for the patient and better control of the position of the teeth in the three planes of space [1]. The straight-wire technique is based on sliding mechanics, in which frictional force plays an essential role. According to some authors, during sliding mechanics, between 12% and 60% of the orthodontic force applied to a tooth is dissipated in the form of static frictional force and orthodontic tooth movement occurs only when the orthodontic force exceeds the existing friction force at the bracket-archwire-ligation system interface [2,3,4,5,6].

The greater the frictional force in the orthodontic system, the more percentage of applied orthodontic force is lost and therefore the actual force transmitted to the teeth decreases [7]. Under these conditions, to overcome the frictional force and initiate the periodontal response, the practitioner must proportionally increase the intensity of the orthodontic force. The use of additional forces can favor the appearance of root resorption and interfere with the process of bone remodeling, causing delay and even limitation of orthodontic movement [8,9]. Furthermore, an excessive orthodontic force changes the balance between the areas of action and reaction, which can further affect the orthodontic anchorage.

Thus, when choosing the components of the fixed orthodontic appliance, it is important to evaluate the variables involved in the variation of frictional force. Mechanical variables include, among other parameters, specific bracket characteristics, such as material and configuration in terms of ligation system—conventional or self-ligating [3,10]. Regardless of the ligation system, the metallic brackets are considered to produce less frictional force compared to ceramic brackets [11]. 

The increasing interest of patients in orthodontic devices that are less visible and can ensure faster favorable results has led to the development of many alternatives to conventional orthodontic appliances, including the aesthetic appearance of brackets and the ligation system. These variables have aroused and still arouse the interest of the orthodontic specialists, especially considering the development of new types of brackets, some of which are promoted precisely by their quality of reducing frictional forces [12]. The combined brackets (ceramic brackets with metallic slots) seem to exhibit lower frictional resistance when compared to ceramic brackets, therefore they may represent an efficient biomechanical alternative [13]. 

In addition, due to the existing COVID-19 pandemic, many professionals advocate the necessity of using dental orthodontic appliances with improved mechanical capabilities, that allow the reduction of total treatment time and the number of required chair side appliance activations [14,15].

The aim of this study was to determine whether there were any differences between the static frictional forces at the orthodontic system interface depending on the bracket material and bracket ligation type.

## 2. Materials and Methods

### 2.1. Sample

The research group consisted of conventional and self-ligating brackets (passive), each type being made of stainless steel and polycrystalline alumina (Table 1). All brackets used were twin, with MBT prescription and 0.022 × 0.028 “slot. 

**Figure 1:**
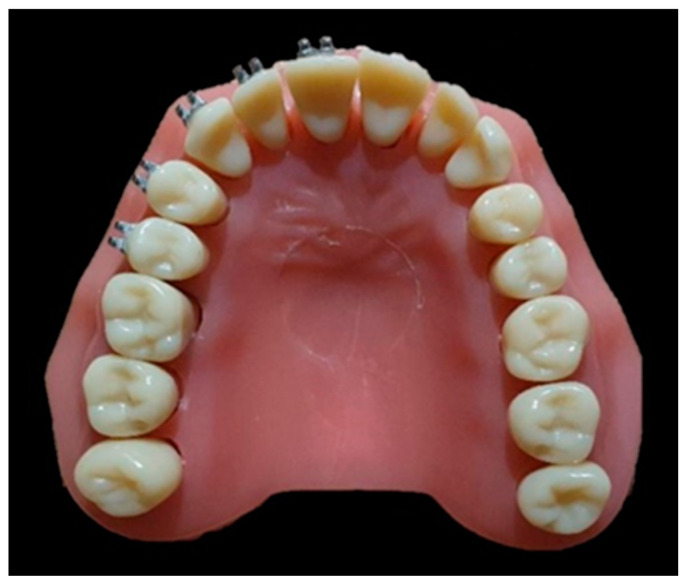
Brackets bonded to standardized model (Spofadent, Fantom Education Models - upper jaw, Kerr Company, Orange, CA, USA).

To simulate the intraoral situation, the brackets were passively bonded on standardized upper arch models (Spofadent Fantom Education Models-upper jaw, Kerr Company, Orange, CA, USA), with orthodontic composite resin (OPAL Bond MV, Ultradent, South Jordan, UT, USA), light-cured for 30 s per bracket (Figure 1). From each bracket category we used 5 brackets-from the upper right central incisor to the upper right second (Figure 1).

**Figure 2:**
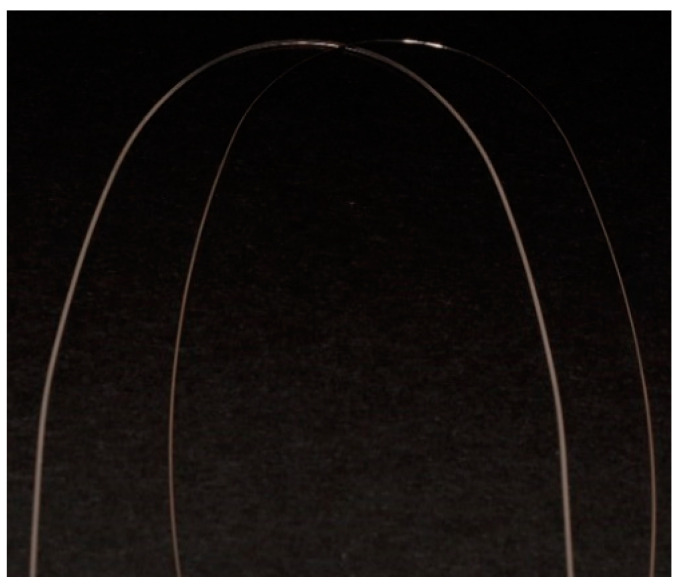
Orthodontic archwires used in the study (AlphaWire, Orthofocus, Bucharest, Romania).

The brackets were tested with ovoid archwires (AlphaWire, Orthofocus, Bucharest, Romania) with different sizes and materials, respectively, 0.016” NiTi and 0.019 × 0.025” SS (Figure 2). We chose these wire dimensions and materials due to their routine use during the initial, respectively, the final phases of orthodontic treatment with fixed appliances, where the bracket slot is 0.022 × 0.028”. Before testing, the brackets and archwires were cleaned with ethanol (70% concentration) and left to dry for one hour, in order to eliminate substances that could influence the results.

**Figure 3:**
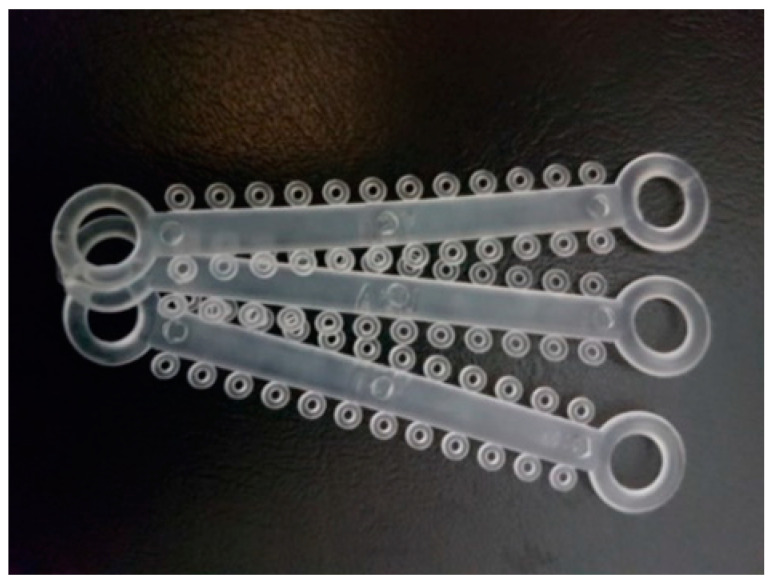
Elastomeric ligatures used in the study (Opal Orthodontics, Ultradent, South Jordan, UT, USA).

For conventional brackets the archwire was fixed by means of transparent elastomeric ligatures (0.12” diameter Opal Orthodontics, Ultradent, South Jordan, UT, USA), applied to the models before each test round (Figure 3).

### 2.2. Testing Protocol

Static friction assessment was carried out with a universal testing machine, HV-500N-S (Schmidt Control Instruments, Hans Schmidt & Co GmbH. Available online: https://www.hans-schmidt.com/en/produkt-details/test-stand-hv-500n/, accessed on 6 December 2021), intended for measuring compression and traction forces, with a maximum capacity of 500 N, from the Faculty of Mechanical Engineering and Mechatronics, University “Politehnica” of Bucharest. 

The testing protocol was also used in previous researches [16,17,18]. The archwire was pulled to the device until the digital distance measuring system signaled its movement, thus electronically identifying the moment of recording the static frictional force. The recorded values were expressed in Newtons (N). In order to obtain conclusive results, the tests were repeated six times for each type of bracket–archwire association and average values were used. Regarding the test conditions, all the experiments were carried out in a dry environment, at a constant temperature of 22.5 ± 5 °C, and the handling of models and testing device belonged to the same team.

Given the variation of frictional force with numerous variables, each type of bracket was also subjected to scanning electron microscopy (SEM) analysis in order to highlight the surface characteristics of the materials of the analyzed brackets. The SEM analysis was performed with the Quanta Inspect F scanning electron microscope (manufacturer FEI-PHILIPS, Netherlands) provided by POLITEHNICA University of Bucharest.

The collected data were centralized and statistically processed using Microsoft Office Excel/Word 2013 and IBM SPSS Statistics v20 (SPSS, Chicago, IL, USA) [19]. The variables were expressed as mean values with standard deviations and tested for distribution using the Shapiro–Wilk test. Independent quantitative variables with parametric distribution were tested using the Student’s *t*-test and those with non-parametric distribution were tested using the Mann–Whitney U test. The level of statistical significance (*p*) was set at a maximum of 0.05.

## 3. Results

The tests generally revealed significant differences between the static frictional forces generated by conventional and self-ligating brackets, regardless of the material from which they were made.

The values of static frictional forces produced at the bracket–arc interface exhibited, according to the Shapiro–Wilk test, a normal distribution (*p* > 0.05), except for the association of conventional brackets with rectangular 0.019 × 0.025” SS archwires. In order to highlight the differences between the frictional forces generated by each type of bracket, the results were grouped into tables according to the archwire used for testing (Table 2 and Table 3).

Results showed that stainless-steel brackets generated significantly lower static frictional forces compared to those made of polycrystalline alumina, for both conventional and self-ligating brackets. These differences were noticed for all conducted tests, with round NiTi (Table 2) and rectangular SS (Table 3) archwires. Descriptively, the largest differences were observed between conventional stainless steel and polycrystalline alumina brackets when combined with 0.019 × 0.025” SS archwires, that fill most of the bracket slot. At the opposite pole, smaller (but still significant) differences were noted between self-ligating stainless steel and polycrystalline alumina brackets tested with thin, flexible 0.016” NiTi archwires.

In this context, we considered the scanning electron microscopy analysis useful, highlighting the possible differences between the morphology of bracket surfaces depending on the material they are made of.

**Figure 4:**
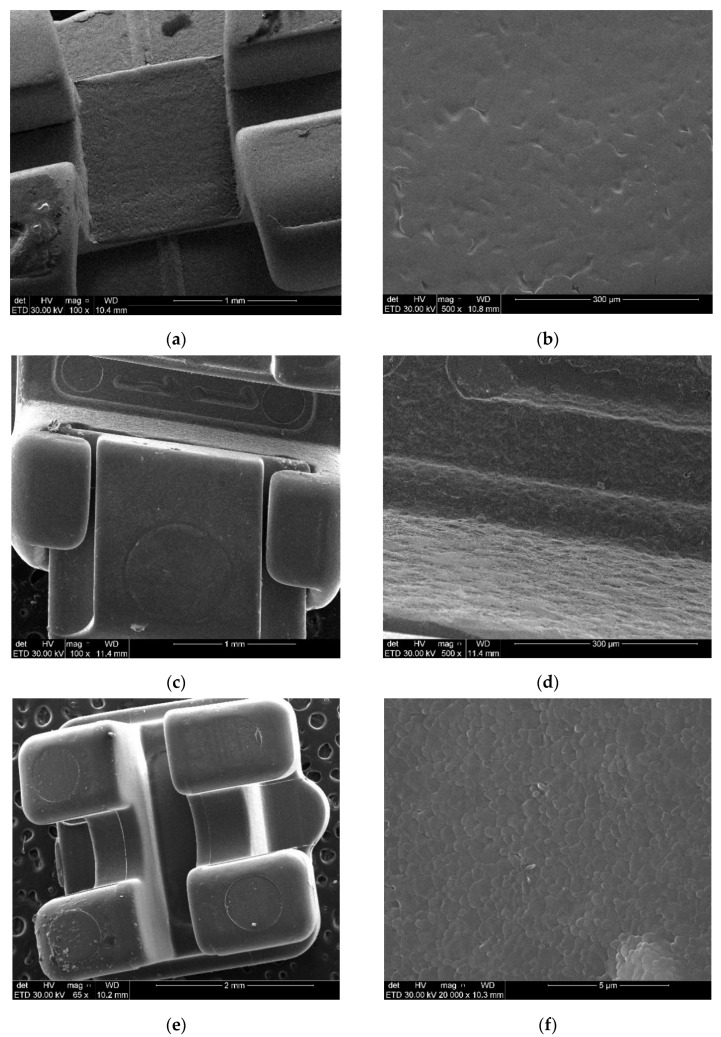

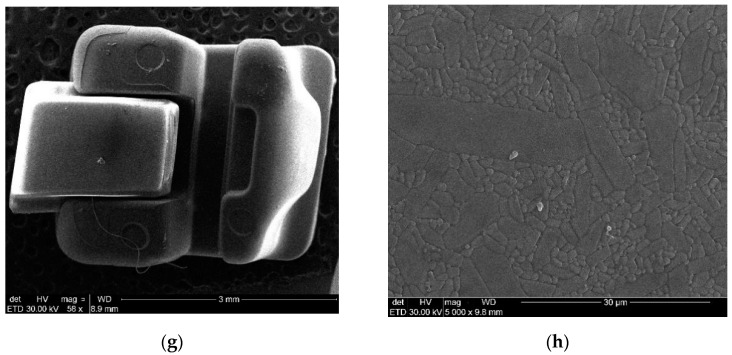
Scanning electron microscopy (SEM) images of stainless steel brackets: (**a**,**b**) conventional brackets; (**c,d**) stainless steel self-ligating brackets; (**e**,**f**) polycrystalline alumina conventional brackets; (**g**,**h**) polycrystalline alumina self-ligating brackets. (**a**) Magnification 100×, (**b**)magnification 500× (**c**) magnification 100×, (**d**) magnification 500×, (**e**) magnification 65×, (**f**) magnification 20,000×, (**g**) magnification 58×, (**h**) magnification 5000×.

Thus, for both types of stainless-steel brackets (Figure 4a–d) the structural uniformity of the surface from the point of view of flatness was highlighted, with parallel lines indicating a correct technological manufacturing process. However, with the increase in magnification, some non-uniformities can be observed that can eventually alter the friction behavior of the material. Regarding the polycrystalline alumina conventional and self-ligating brackets (Figure 4e–h) the characteristic appearance of this type of material was noted [20]. Microcrystals of different sizes and orientations, quasi-uniform distribution of alumina granules, compactly joined into a common matrix, resulting in an increase of surface roughness, may be responsible for augmenting the value of the friction force at the bracket–archwire interface.

In terms of bracket ligation method, stainless steel self-ligating brackets in combination with 0.016” NiTi archwires produced significantly lower frictional forces than conventional metal brackets tested with the same type of archwire. However, for tests conducted with 0.019 × 0.025” SS archwires (Table 3), self-ligating and conventional metal brackets produced almost equal frictional forces (3.85 ± 0.288 N versus 3.883 ± 0.1 N); these values were not statistically different when compared with Student’s *t*-test (*p* = 0.785).

There were also differences between the static frictional forces produced by conventional and self-ligating polycrystalline alumina brackets. Self-ligating polycrystalline alumina brackets produced significantly lower static frictional forces than conventional ones regardless of the orthodontic archwire used. Descriptively, from the point of view of frictional forces, the biggest differences between self-ligation and conventional ligation were observed for polycrystalline alumina.

## 4. Discussion

Orthodontic forces adaptation to clinical requirements promotes an optimal tissue response and efficient tooth movement. During mechanics involving the movement of the bracket along the archwire/ archwire along the bracket, the frictional force at the bracket–archwire interface can prevent the optimal transmission of forces to the periodontal structures. Therefore, a thorough understanding of the variables that influence the frictional force is important, so that the physician can properly adjust the intensity of the orthodontic force for optimal tooth movement [3].

After interpreting the results of the experiments, we noticed that the static friction force at the bracket-archwire interface varied with bracket material, but also varies between conventional and self-ligating brackets.

The metal brackets, either conventional or self-ligating, have generated the lowest static frictional forces, regardless of the material or size of the orthodontic archwire with which they were tested. Analyzing quantitatively, for conventional brackets, those made of polycrystalline alumina were associated with frictional forces 1.87 times (for tests conducted with 0.016” NiTi archwires), respectively, 2.27 times (for tests conducted with 0.019 × 0.025” SS archwires) higher than the metallic ones. In the case of self-ligating brackets, ceramic brackets generated frictional forces 1.53 times higher than stainless steel ones, regardless of the tested archwires. The results can be explained in terms of the mechanical characteristics of metal brackets, which allow a superior finishing, with lower surface roughness of the bracket slot thus favoring orthodontic mechanics [21,22,23]. These findings are also confirmed by the SEM analysis performed in this study. The results of our research are in agreement with most of the specialized studies that evaluate comparatively aesthetic and metallic brackets. In this context there is an almost unanimous opinion that stainless steel brackets are associated with lower frictional forces than aesthetic brackets [21,22,24].

De Franco et al. quoted by Sukh et al. (2013) found that in the vast majority of tests the metal brackets generated the lowest frictional forces [23]. Similar results were obtained by Pattan et al. (2014), which highlighted that frictional forces were smaller for metal brackets regardless of slot size or test conditions [25]. Higher static frictional forces obtained by testing the ceramic brackets can be explained by the mechanical properties of polycrystalline alumina [23,26]. Thus, the hardness, rigidity and surface roughness, with granulations and depressions of ceramic materials, as proved by the SEM analysis, are considered the main determinants in increasing the value of friction [23].

The results obtained in our research are consistent with findings from other studies, which indicate that the material, and moreover, its surface characteristics significantly influence the variation of the friction force [27,28,29,30]. This phenomenon can be explained by taking into account the difference between the apparent and the true contact area between two surfaces. The apparent surface is the geometric area of contact, which is “ideal” because it is practically impossible to obtain by processing. In reality, the contact between the two bodies is made through the micro-contacts of the irregularities, which represent the true/effective surface [31]. Thus, the effective surface, dependent on surface roughness, is the one that influences the friction force both directly and indirectly [28,30]. The direct link can be explained by Coulomb’s theory, which postulates that frictional forces depend on the irregularities of the surfaces in contact. It follows, by virtue of this theory, that the finer the surfaces are machined/ processed, the lower the frictional force between the two bodies [32]. Indirectly, the number of irregularities on the surface increases as the effective surface increases, and with it the adhesion rate of bacteria on stainless steel surfaces [28,30]. Bacterial adhesion favors accumulation of microbial plaque on orthodontic appliance surfaces, which in the context of poor oral hygiene turns into hard calcified deposits. This debris at the interface of the orthodontic system (bracket-arch-ligature) not only poses oral health risks but also influences the value of friction during treatment [33,34]. 

Over time, researchers have been concerned with this issue, both in terms of bracket material selection and surface treatment. As stated previously, stainless steel brackets generate the lowest frictional forces, but it is important to mention that their mechanical behavior depends also on differences in manufacturing process. It was reported that sintered stainless steel brackets were associated with less friction than cast ones, due to their smoother finishing [29]. Still, demand for aesthetic components of orthodontic appliances has led to the search for alternatives to metal brackets. Composite, polycarbonate brackets lost their popularity because of unsatisfactory mechanical properties. Lining the composite bracket slot with a thin layer of stainless steel and the presence of an oxide film on the metal slot played a role in decreasing their static friction to levels comparable to stainless steel brackets [29]. Ceramic brackets, however, are used on a larger scale because of their superior mechanical qualities. Differences were also reported between different types of ceramic brackets. Some studies show that monocrystalline sapphire monocrystalline alumina brackets are associated with less (or comparable) frictional forces than polycrystalline alumina brackets [35,36]. Regarding this aspect, most opinions in the literature remain conflicting, other research reporting higher friction forces for monocrystalline than polycrystalline alumina brackets [37,38,39].

Self-ligating brackets have received a lot of attention lately and their use has increased considerably. Some authors argue that most of the benefits associated with this ligation system—shorter treatment duration, improved expansion of dental arches and reduction of incisor proclination during treatment mechanics—are due to the reduced frictional forces by which they are promoted [40,41,42,43,44,45,46,47,48,49,50,51]. However, this topic is a controversial one, as there are many authors who have different opinions. They point out that there is insufficient scientific evidence to support the effectiveness of self-ligating systems [52,53,54].

In our study, self-ligating brackets generally produced significantly lower static frictional forces than conventional ones. This observation is in line with a significant amount of research on this topic [7,55,56]. From a quantitative perspective, our results showed that in tests performed with 0.016” NiTi archwires, frictional forces were four times higher for conventional metal brackets compared to the self-ligating metal brackets. The difference between the results was even greater in the case of ceramic brackets, those with conventional ligation system being associated with frictional forces 5.82 times higher than self-ligating ones. The significant reduction in static frictional force, especially in case of thin and flexible wires, is mainly due to the elimination of elastomeric ligatures. The fastening system of the passive self-ligating brackets acts as a fourth (mobile) wall of the bracket, creating a passive lumen to hold the archwire in the bracket slot, without actively exercising forces on the wire.

However, it should be noted that our research proved that in case of tests performed with the rectangular archwires, which almost entirely filled the bracket slot, no statistically significant differences were noticed between the frictional forces occurring in conventional and self-ligating stainless-steel brackets. As the treatment progresses, rectangular archwires are used so that fine dental movements are performed. More and more contact points between the archwire and the bracket appear, eliminating the “play” between the two, which causes the frictional forces to increase significantly. This observation is consistent with other studies in the scientific literature [57]. Similar results were obtained in the study by Henao and Kusy (2004). They found that self-ligating brackets produced lower frictional forces than conventional ones when combined with 0.014” archwires, while for the use of 0.016 × 0.022” and 0.019 × 0.025” archwires the values of the frictional forces did not differ significantly [56].

Thus, it can be stated that the efficiency of the self-ligating system is largely present when used with thinner, flexible archwires. In this context, a meta-analysis conducted in 2015 highlighted that no statistically significant differences were observed between conventional and self-ligating brackets in terms of closing speed of edentulous spaces [58]. Nevertheless, with regard to polycrystalline alumina brackets, the self-ligating brackets resulted in lower frictional forces than the conventional ones. Quantitatively, for the use of 0.019 × 0.025” SS archwires, the values of the frictional forces were 1.5 times lower in the case of ceramic self-ligating brackets compared to conventional ceramic ones. Thus, in the situation where it is necessary to apply a fixed aesthetic orthodontic appliance, it would be more appropriate to choose the self-ligating alternative.

### Limitations of the Study

The results of this study should be viewed in light of the limitations associated with all in vitro studies. It is important to note that tests performed in laboratory conditions cannot fully simulate the intraoral situation, knowing that the biological variables greatly influence the frictional forces. Furthermore, the handling of the testing machine was done manually, which can lead to measurement errors. Another source of inaccuracy can be attributed to the bracket support models, which, although standardized, may have small manufacturing differences. Although the literature includes multiple studies on friction in orthodontics, the lack of homogeneity of the methodology imposes certain reservations regarding the comparative analysis of the results.

## 5. Conclusions

Study results revealed significant differences in static frictional forces at the bracket-archwire interface between conventional and self-ligating brackets and between metal and ceramic brackets.

Stainless steel conventional/self-ligating brackets have produced lower static frictional forces than those made of polycrystalline alumina. So, from the perspective of an orthodontic system with low frictional forces, metal brackets are preferable to aesthetic ones.

Self-ligating brackets generally produced lower static frictional forces than conventional brackets. However, the reduction of frictional forces is more marked in the first stages of treatment, when thin, flexible orthodontic archwires are used. As the treatment progresses and the archwire fills the slot, frictional forces produced by conventional and self-ligating metal brackets are similar. Nevertheless, in the case of polycrystalline alumina brackets, the results suggested that the self-ligating bracket type allows a reduction in frictional forces even in advanced stages of treatment compared to conventionally ligation.

## Figures and Tables

**Table 1:** Types of brackets included in the study.

Brackets			
Conventional		Self-Ligated	
stainless steel (Mini Sprint, Forestadent, Pforzheim, Germany)	polycrystalline alumina (Discovery Pearl, Dentaurum, Ispringen, Germany)	stainless steel (Damon Q, Ormco, Brea, CA, USA)	polycrystalline alumina (Damon Clear, Ormco, Brea, CA, USA)

**Table 2:** Comparison of static frictional forces (mean values) measured for brackets with 0.016” NiTi archwires.

0.016” NiTi Archwire	Stainless Steel Brackets	Polycrystalline Alumina Brackets	*p*
**Conventional brackets**	1.65 ± 0.258 N	3.087 ± 1.054 N	<0.001 *^,1^
**Self-ligating brackets**	0.35 ± 0.115 N	0.536 ± 0.126 N	<0.05 *^,1^
***p***	<0.001 *^,1^	<0.001 *^,1^	

* statistically significant results, ^1^ Student’s *t*-test, Newtons (N).

**Table 3:** Comparison of static frictional forces (mean values) measured for brackets with 0.019 × 0.025” SS archwires.

0.019 × 0.025” SS Archwire	Stainless Steel Brackets	Polycrystalline Alumina Brackets	*p*
**Conventional brackets**	3.85 ± 0.288 N	8.741 ± 1.299 N	<0.001 *^,2^
**Self-ligating brackets**	3.883 ± 0.1 N	5.933 ± 0.622 N	<0.001 *^,1^
***p***	0.785 ^1^	<0.001 *^,1^	

* statistically significant results, ^1^ Student’s *t*-test, ^2^ Mann–Whitney U, Newtons (N).

## Data Availability

The data presented in this study are available on request from the corresponding author.
